# Evaluation of the Accuracy of Working Casts Fabricated by Four Implant Impression Techniques in Parallel and Nonparallel Implant Configurations

**DOI:** 10.1055/s-0045-1809980

**Published:** 2025-09-08

**Authors:** Ahmad Al Jaghsi, Dinesh Rokaya

**Affiliations:** 1 Clinical Sciences Department, College of Dentistry, Ajman University, Ajman, United Arab Emirates; 2 Center of Medical and Bio-Allied Health Sciences Research, Ajman University, Ajman, United Arab Emirates; 3 Department of Removable Prosthodontics, Damascus University, Damascus, Syria; 4 Department of Prosthodontics, Gerodontology and Dental Materials, Greifswald University Medicine, Greifswald, Germany

**Keywords:** dental implants, implant impressions, parallel implants, nonparallel implants

## Abstract

**Objective:**

The implant impression technique plays a critical role in determining the accuracy of implant working casts. This laboratory study aimed to evaluate and compare the accuracy of working casts fabricated using four different impression techniques in both parallel and nonparallel implant configurations.

**Materials and Methods:**

An aluminum master model was made to simulate a mandibular dental arch. Impressions of the dental implants were made using four techniques within an incubator: (A) unsplinted tapered impression copings; (B) unsplinted squared impression copings; (C) splinted squared impression copings with dental floss supported with self-curing acrylic resin; and (D) splinted squared impression copings with prefabricated self-curing acrylic resin bars. Measurements were made using a universal measuring microscope in the *x* - and *y* -dimensions. The mean deviation index was calculated and compared. Statistical analysis was done using SPSS version 23. Comparisons of the distance deviation index in various groups were conducted using one-way analysis of variance. Furthermore, comparisons of the distance deviation index in nonparallel implants in various techniques were performed using the independent *t* -test. A *p* -value of 0.05 was considered a significant difference.

**Results:**

For the parallel implants, the distance deviation index difference between the techniques was not significant. In the case of nonparallel implants, there was statistically significant difference only between the technique C versus technique D ( *p* =  0.019). All the impression techniques showed statistically significant differences between parallel versus nonparallel implant placement in favor of the parallel. Overall, technique D demonstrated the best results for both parallel and nonparallel implant placements when compared with other techniques under the same conditions.

**Conclusion:**

The impression technique affects the accuracy of the implant impression. Within the limitations of this study, impression transfer copings splinted with dental floss and reinforced with self-cure acrylic resin are not recommended, particularly when the implants are not parallel. In the case of parallel implants, there appears to be no statistically significant benefit from splinting transfer impression copings.

## Introduction

Currently, dental implants are commonly used for the rehabilitation of patients with complete and partially edentulous arches. 1 2 Considering this, the accuracy of the impression of dental implants is a critical factor for implant prosthesis success. Numerous factors affect the accuracy of implant restorations, such as impression materials and techniques, 3 4 5 implant angulations, 6 abutment types, 7 and prosthetic connection features. 3 In addition, edentulous space type, bone quality, surgical guide, and surgery protocol affect the implant placement. 8 All these factors affect the success of prosthetic restorations. 9

The implant placement can be either parallel or tilted (nonparallel). 10 11 12 It is found that the stress is higher in the tilted implants. Naini et al 10 conducted a study to evaluate stress concentration in peri-implant bone in two different configurations: the distal implants tilted (model A) or four parallel implants (model S) and hybrid superstructures, and they found that during posterior loading, the tilted posterior implants were subjected to higher stresses in every condition but lower stress concentrations around the anterior implants of model A. In addition, Vojdani et al 11 compared the accuracy of implant impressions using three impression materials in both parallel and nonparallel implant positions and they found that in parallel conditions, the type of impression material does not affect the accuracy of the implant impressions; however, in nonparallel conditions, polyvinyl siloxane shows a better choice, followed by vinyl siloxane ether and polyether, respectively.

Furthermore, the implants can be splinted or unsplinted and it shows that the splinted technique demonstrated higher accuracy compared with the unsplinted technique. 13 At present, various digital aids are used in clinical dentistry, including implant placement and planning. 14 15 The patients prefer the digital impression over the conventional impression due to convenience, time, and comfort. 16 17 18 For the implant prosthesis, the accuracy of the implant impression is an important step. 19 20 The accuracy of digital impressions using intraoral scanners is acceptable for single crowns and short-span implant restorations. 21 22 23 But the digital impressions may show distortion for more than two implants in the long-span prosthesis. 24 25 Hence, while making the impression for long-span implant-supported restorations, the use of digital impression presents some difficulties and subsequently affects the fit of the implant restoration. 26 Various studies show that the conventional impression technique shows more accuracy than the digital impression technique, especially for long-span prostheses. 24 25 27 28 Hence, in full arch impression, the digital impression can show some error, hence, one needs to consider this when making an impression for long-span prosthesis.

In addition, there is limited information about the accuracy of impression techniques in parallel versus nonparallel implants in the complete edentulous arch. 29 This study aimed to evaluate and compare the accuracy of four impression techniques in angulated implants and the effect of the parallelism of the implants on the accuracy of the working model.

## Materials and Methods

### Metal Model

**Fig. 1:**
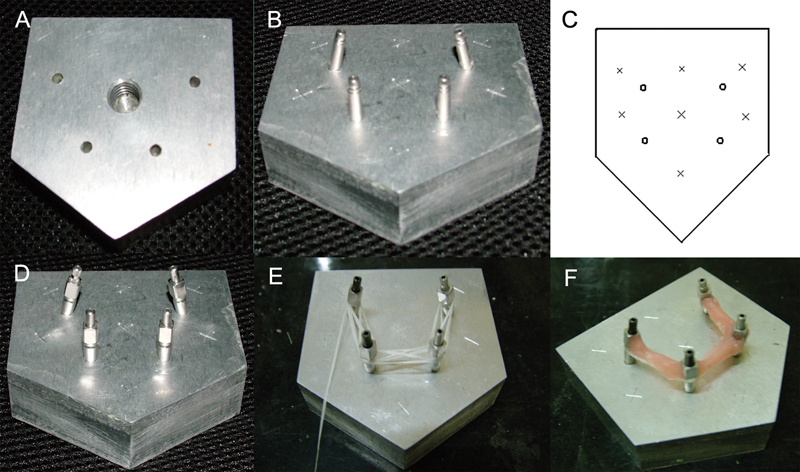
Master model and study groups. ( **A** ) The lower face of the aluminum master model. ( **B** ) Technique A: Transfer impression copings. ( **C** ) X-shape reference points. ( **D** ) Technique B: Square unsplinted impression copings with long fixation screw. ( **E** ) Technique C: The impression copings were splinted with dental floss and self-curing acrylic resin. ( **F** ) Technique D: The impression posts were splinted with prefabricated acrylic bars and secured to the square impression copings with a small amount of acrylic resin.

An aluminum master model was made to simulate a mandibular dental arch ( Fig. 1A ) with four holes to accommodate four implants. The holes were distributed like how implants are placed in the lower jaw. The two holes in the front were made parallel and the two holes at the back were inclined 15 degrees inward (lingually) to simulate the lingual tilt in the posterior regions of the lower jaw ( Fig. 1B ). Then, seven X-shape reference points ( Fig. 1C ) were placed in healing caps with precisely defined centers placed over the implants to make measurements at later stages as described previously. 29 Three individual trays (one closed and two open trays) were made from self-curing acrylic. Stoppers were placed inside the trays to unify the thickness of the impression material across all impressions; the protocol was the one utilized by Herbst et al. 29

### Impression and Working Model Fabrication

The study sample consisted of 64 impressions across four techniques (A, B, C, and D) as shown in Table 1 . Two impressions were discarded due to deformation, resulting in 62 gypsum models being used for analysis. Technique A was only a closed-tray technique, whereas all other techniques (B, C, and D) were open-tray techniques ( Table 1 ). The implants: two anterior parallel implants (implants 1 and 2) and two posterior implants (implants 3 and 4) inclined at 15 degrees inward (lingual). For each gypsum model, 56 readings were recorded and a total of 3,472 readings were recorded for all gypsum models as shown in Table 2 .

**Table 1:** Various impression techniques used for impression in this study

Techniques ( *N* = 62)	Details
Technique A ( *n* = 16)	A closed-tray with tapered unsplinted transfer impression copings.
Technique B ( *n* = 15)	An open-tray with square unsplinted transfer impression copings
Technique C ( *n* = 16)	Open-tray with square transfer impression copings splinted by dental floss and self-curing acrylic resin
Technique D ( *n* = 15)	Open-tray with transfer impression copings splinted by prefabricated acrylic bars and self-curing acrylic resin

**Table 2:** Descriptive statistics of the distance deviation index in various techniques

Impression techniques	Number of measurements	Mean	SD	SE
Technique A (closed-tray, unsplinted copings)	64	0.1319	0.1633	0.0204
Technique B (open-tray, unsplinted copings)	60	0.1136	0.1131	0.0146
Technique C (Open-tray, splinted copings by dental floss and self-curing acrylic resin)	64	0.1613	0.1687	0.0211
Technique D (Open-tray, splinted copings by prefabricated acrylic bars)	60	0.0909	0.0732	0.0095

Abbreviations: SD, standard deviation; SE, standard error of the mean.

In technique A, the impression copings were fixed to the implants, and the impression was taken using a closed-tray impression technique ( Fig. 1D ). After the set, the impression was removed from the metal cast, leaving the impression copings on the implant. The impression coping was removed from the metal cast (master model), the implant replica was attached to the coping, then it was repositioned in the impression, and the gypsum was poured. In technique B, an open impression was used where the square transfer impression coping was placed on the model using a long fixation screw ( Fig. 1D ). A window was made in the impression at the location of the screw so it can be seen through it. After the material was set, the impression was removed after loosening the screw leaving the impression post in place within the impression material. In technique C, the impression posts were attached with dental floss (Oral B Essential Floss, Procter & Gamble, United States) ( Fig. 1E ), and self-curing acrylic (Dura Lay, Reliance Dental Manufacturing Co, Worth, Illinois, United States) was placed. In technique D, an open-tray impression was used where square impression copings were connected by prefabricated acrylic bars with an average thickness of 5 mm, with a small amount of acrylic ( Fig. 1F ).

The impression was made with polyvinyl siloxane (Dimension Penta H; 3M ESPE, Seefeld, Germany) using the double impression technique (single-stage impression). The heavy body was mixed and placed inside the tray and the light body was injected on the heavy body and around the impression copings, according to the manufacturer's instructions. All impressions were taken in an incubator (Taba l, Tsugami, Japan) at 37°C and 98% humidity to simulate the oral environment. The impressions were poured with gypsum according to the manufacturer's instructions. The metal model was placed in the incubator for 10 minutes before taking the impression to become homogeneous at 37°C and 98% humidity. Then, gypsum for model fabrication (Moldano, Heraeus Kulzer GmbH & Co., Germany) was used to approximate the working conditions in our laboratory. After pouring, all gypsum models were kept at room temperature for 24 hours before doing measurements.

### Measurements and Calculations

**Fig. 2:**
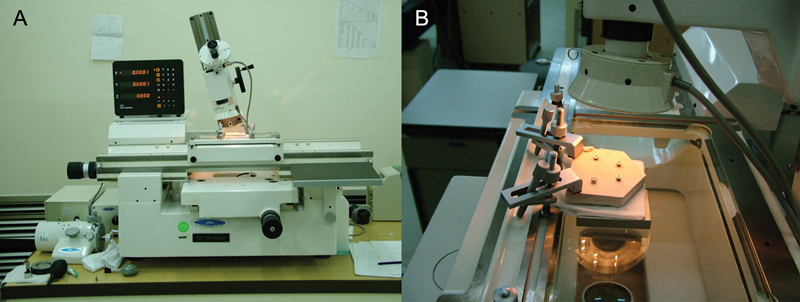
( **A** ) Universal measuring microscope and ( **B** ) Close-up view of the model for the measurement of the dimensions.

The healing cap was placed on the implants in the master model, and then the dimensions were measured between the center of each healing cap and the seven points engraved on the master model using a universal measuring microscope with an accuracy of 0.1 micrometer (UM2D, Tsugami, Japan) ( Fig. 2 ). Then, these caps were transferred to the gypsum models to take the measurements so that the healing cap no. (1) was placed on implant no. (1), and so on. The gypsum models were fixed on a base with two arms resting on the back corners of the model to prevent its movement. The coordinates ( *X* points) (A, B, C, D, E, F, G) located on the surface of the gypsum model were measured for each of the implants (1, 2, 3, 4) under the constant working conditions of temperature (20 ± 1°C) and relative humidity (55 ± 5%). Then, the direct distance between the points and the implants was estimated based on the Pythagorean theorem. The readings were taken by the researcher with the help of laboratory technicians.

The accuracy of the readings was evaluated by measuring the distance between two points by a single reader more than 30 times, and the maximum difference did not exceed 0.0003, per manufacturer recommendation. The researcher and laboratory technician were asked to take the measurement between two points 30 times, and the maximum recorded difference was 0.0028 mm, which is acceptable per the manufacturer's recommendation. All the measurements were recorded with an accuracy of 0.0001 mm as mentioned by previous studies on different implant elements. 30

**Fig. 3:**
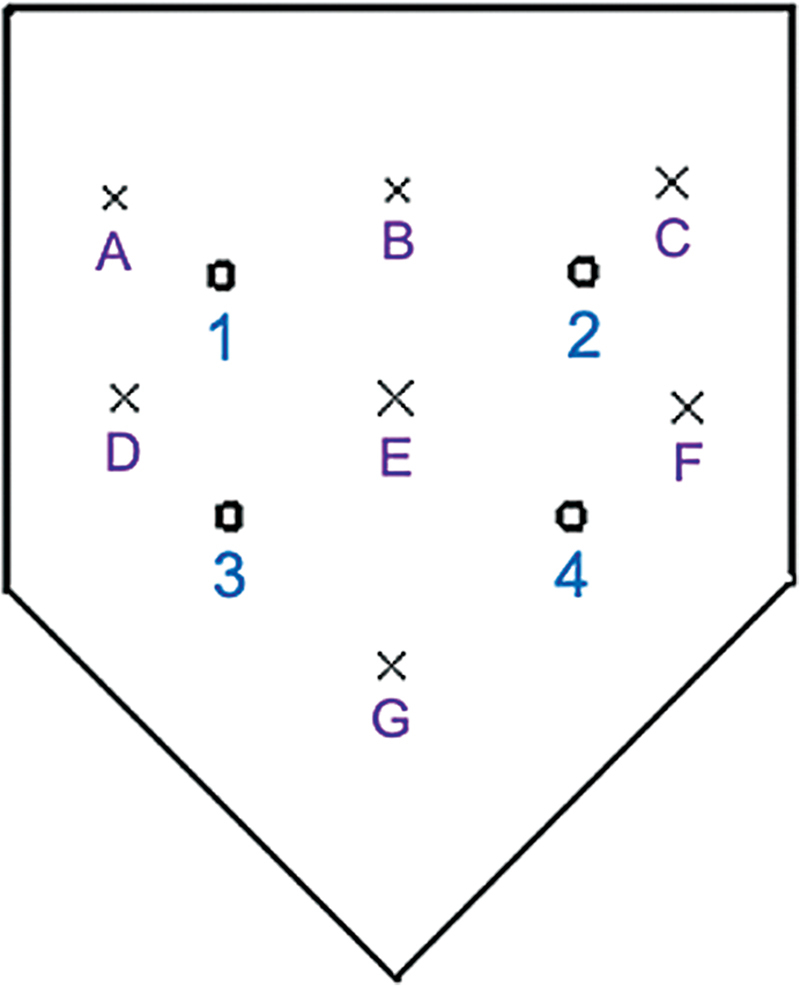
Coordinates (X points) (A, B, C, D, E, F, G) located on the surface of the gypsum models were measured for each of the implants (1, 2, 3, 4).

The master model was measured three times and then the mean was calculated. The distance between the seven points on the model was measured ( Fig. 3 ). For each of the four implants (after placing the healing cap on it), the distance is calculated as the total distance of the implant as follows:

TD (total distance) of implant 1 = Distance from Implant 1 to point A + Distance from Implant 1 to point B + Distance from Implant 1 to point C + Distance from Implant 1 to point D + Distance from Implant 1 to point E + Distance from Implant 1 to point F + Distance from Implant 1 to point G

Similarly, the healing caps were then transferred to the gypsum model and measurements were made to compare with the values of the master model. By calculating the value of the distance, the deviation index for each implant was calculated from the following equation:

Distance deviation index for implant 1 = [Total distance value in the Master model – value in the gypsum model | Total distance value in the Master model] × 100. 29

### Statistical Analysis

All data were entered in Excel and then into SPSS version 23 analysis software. Descriptive statistics (mean, standard deviation [SD], and standard error of the mean [SE]) were computed to summarize the data. Comparisons of the distance deviation index among the four techniques were done using one-way analysis of variance with post hoc analysis. Furthermore, comparisons of the distance deviation index in nonparallel implants among the four techniques were performed using the independent *t* -test. A *p* -value of 0.05 was considered a significant level.

## Results

### Effect of Different Impression Techniques on Dimensional Accuracy as Measured by Deviation Index

The deformity is defined as the relative movement of a specific point or a group of points relative to a point or several reference points. 31 In this study, the fixed reference points were marked as X (A, B, C, D, E, F, G), while the variable points were the implants, labeled 1, 2, 3, and 4. Table 2 shows the descriptive statistics, mean, SD, and SE of the distance deviation index according to the impression technique and provides insight into the relative performance of each technique. Technique C showed the highest mean deviation index compared with techniques A, B, and D.

Furthermore, Table 3 shows the group comparisons of the distance deviation index in various techniques. It showed that there was a significant difference among the groups ( *p* = 0.033). Furthermore, the mean differences of the distance deviation index in various techniques and their multiple comparisons among the four techniques with *p* -values between each pair are shown in Table 4 . It was found that statistically significant differences existed for the distance deviation index between various technique pairs, including technique A versus technique C ( *p* -value = 0.027), technique B versus technique C ( *p* -value = 0.027), and technique B versus technique D ( *p* -value = 0.027). The comparisons like technique A versus technique B and technique A versus technique D showed no significant differences ( *p* -value > 0.05). Overall, technique D outperforms the other techniques in most comparisons.

**Table 3:** Comparisons of the distance deviation index in various techniques

Impression techniques	Sum of squares	Degree of freedom	Variance	*F*	*p* -Value
Between groups	0.165	3	0.055	2.948	0.033 a
Within groups	4.543	244	0.019		

Note: Statistical analysis was done using one-way analysis of variance (ANOVA).

a Significant difference at *p* -value of 0.05.

**Table 4:** Multiple comparisons of the distance deviation index in various techniques

Impression techniques	Comparison group	Mean difference	SE	*p* -Value
Technique A	Technique B	0.0183	0.02	1.000
	Technique C	−0.0294	0.02	0.027 a
	Technique D	0.0410	0.02	0.577
Technique B	Technique C	0.0227	0.02	0.027 a
	Technique D	0.0703	0.02	0.027 a
Technique C	Technique D	0.0703	0.02	0.027 a

Abbreviation: SE, standard error of the mean difference.

Note: Statistical analysis was done using one-way analysis of variance (ANOVA) with post hoc analysis.

a Significant difference at *p* -value of 0.05.

### Influence of Implant Parallelism on Dimensional Accuracy as Reflected by the Deviation Index

Table 5 shows the group comparison of the distance deviation index in nonparallel implants in various techniques. It showed that there was a significant difference among the groups in nonparallel implants ( *p* -value = 0.027). In the case of nonparallel implants, there was a statistically significant difference in the distance deviation index only between technique C versus technique D ( *p* = 0.019) as shown in Table 6 .

**Table 5:** Group comparison of the distance deviation index in parallel and nonparallel implants

Implant parallelism	Impression techniques	Sum of squares	Degree of freedom	Variance	*F*	*p* -Value
Parallel	Between groups	0.009	3	0.003	1.100	0.352
	Within groups	0.324	120	0.003		
Nonparallel	Between groups	0.260	3	0.087	3.154	0.027 a
	Within groups	3.302	120	0.028		

Note: Statistical analysis was done using an independent *t* -test.

a Significant difference at *p* -value 0.05.

**Table 6:** Multiple comparisons of the distance deviation index in nonparallel implants in various techniques

Implant parallelism	Impression techniques	Comparison group	Mean difference	SE	*p* -Value
Nonparallel	Technique A	Technique B	0.0182	0.042	1.000
		Technique C	−0.0661	0.041	0.680
		Technique D	0.0608	0.042	0.911
	Technique B	Technique C	−0.0844	0.042	0.285
		Technique D	0.0426	0.043	1.000
	Technique C	Technique D	0.1269	0.042	0.019 a

Abbreviation: SE, standard error of the mean difference.

Note: Statistical analysis was done using an independent *t* -test.

a Significant difference at *p* -value of 0.05.

Similarly, Table 7 shows the comparison of the distance deviation index in parallel and nonparallel implants across various techniques. All the impression techniques showed statistically significant differences between parallel versus nonparallel implant placement in favor of the parallel. Overall, technique D demonstrated the best results for both parallel and nonparallel implant placements when compared with other techniques under the same conditions.

**Table 7:** Comparison of the distance deviation index in parallel and nonparallel implants in various techniques in groups

Impression technique	Implant parallelism	Mean	Mean difference	SE	*p* -Value
Technique A	Parallel	0.0793	−0.1052	0.0389	0.009 a
	Nonparallel	0.1845			
Technique B	Parallel	0.0611	−0.1051	0.0260	< 0.0001 a
	Nonparallel	0.1662			
Technique C	Parallel	0.0720	−0.1786	0.0360	< 0.0001 a
	Nonparallel	0.2506			
Technique D	Parallel	0.0096	−0.1141	0.0170	< 0.0001 a
	Nonparallel	0.1237			

Abbreviation: SE, standard error of the mean difference.

Note: Statistical analysis was done using an independent *t* -test.

a Significant difference at *p* -value of 0.05.

## Discussion

The present laboratory study evaluated the impact of different impression techniques and implant parallelism on the working casts, with the aim of determining the most accurate method for replicating implant positions. The results demonstrated that technique D yielded the best outcomes for both parallel and nonparallel implant placements when compared with other tested techniques. Interestingly, when implants were placed in a parallel configuration, the study was unable to detect any statistically significant differences between the impression techniques, suggesting that implant parallelism may reduce the technique sensitivity and overall influence on cast accuracy.

An implant impression is crucial to fabricate an implant restoration on a working model that precisely replicates the implant position in the patient's mouth. 32 Splinting the impression posts improves the accuracy of the impression by fixing the impression posts and preventing their movement within the impression material during the different stages of cast fabrication. 33 34 This technique accurately replicates the spatial position of the implant to the model as it exists in the patient's mouth. 35 36 37 Furthermore, while making the implant impression, splinting the implant components using the open-tray method increases the impression accuracy. 38 39 However, Humphries et al 40 evaluated three impression techniques using polyvinyl siloxane and found no significant difference between splinted and nonsplinted open-tray impression methods. The closed-tray impression technique, utilizing tapered impression copings, demonstrated the highest accuracy. However, all three methods were generally capable of producing clinically acceptable impressions. But still, clinicians support that splinting the impression posts improves the accuracy of the implant impression. For the splinting of implant components, Duralay acrylic is commonly used. Additionally, splints made with acrylic resin showed more accuracy than those made with light-cured composite resins. 41 Hariharan et al 42 showed that splinting with acrylic resin is more accurate than the nonsplint method. Furthermore, the concept of attaching impression posts using self-cure acrylic supported on dental floss was recommended previously. 43 However, this technique is relatively complex, requires time and effort, and may be inaccurate. This requires bonding with acrylic to make the impression more accurate with less distortion. 44 Spector et al 44 conducted a study on a model containing six parallel implants and compared the indirect method with and without bonding, and they found that no method was free of distortion. Similarly, Mojon et al 45 conducted a study to estimate the effect of the amount of acrylic on the accuracy of the impression and they found that the larger the amount of acrylic used to bind the impression copings, the greater the distortion. The distortion is due to the shrinkage of the hardened acrylic. 45 46 Furthermore, Mojon et al 45 reported that the shrinkage within the first 24 hours reaches 7.9%, with approximately 80% of this shrinkage occurring within the first 17 minutes after mixing at room temperature. Hence, need to consider this while splinting, but still, it is a preferred material for chairside splinting.

For the full arch implant prosthesis, conventional impressions are used due to accuracy concerns.

A recent study by Jasim et al 28 compared the accuracy of implant-level conventional and digital impressions for atrophied maxillary ridges: (1) conventional (splinted open-tray) impression technique and (2) digital impression technique. The accuracy was measured between two impression techniques using *in vitro* (two-dimensional [2D] and three-dimensional [3D]) and *in vivo* methods. The 2D methods include measurement of the difference in linear distances, whereas 3D deviations were measured by the superimposition of STL files using the Geomagic software. The degree of framework misfits of final restorations was assessed using the single screw in the two impression techniques. It was found that digital impressions showed significantly higher deviation in 2D distances than conventional impressions. Similarly, digital impressions recorded significantly higher 3D deviation for all scan bodies than conventional impressions. And, digital impressions recorded a significantly higher incidence of nonpassive frameworks and framework misfits compared with conventional impressions. And they concluded that the conventional impression technique showed greater accuracy than the digital for the full-arch fixed restorations on inclined implants in the maxillary arch.

Similarly, a study by Elashry et al 47 studied the accuracy of the digital impression compared with the conventional implant impression techniques in bilateral distal extension cases in the mandibular arch using 32 implants. The patients underwent two implant-level impression techniques: digital implant impressions with TRIOS 3 Shape intraoral scanner and conventional open-tray impressions (splinted pick-up) and the accuracy of impressions was evaluated using the 3D superimposition analysis of STL files. Subsequently, the scan bodies were segmented using the Gom Inspect software to measure 3D deviations. It showed that higher positional and angular deviations were shown toward distal scan bodies compared with mesial ones for both impression techniques, but the difference was not significant. They concluded that splinted open-tray conventional impression and intraoral scanning implant impression techniques showed comparable accuracy. Similarly, another study by Palantza et al 48 compared the accuracy of full-arch conventional implant impressions using two different materials (silicone and polyether) to full-arch digital implant impressions produced from two intraoral scanners (Trios vs. Heron). They found that the accuracy of mandibular implant impressions was influenced both by the impression technique (conventional vs. digital) and the impression material used (silicone vs. polyether), or the intraoral scanner. In terms of “trueness,” silicone showed the highest accuracy, followed by polyether and Trios, but the differences between the three groups were not significant. Heron showed lower accuracy results in all measurements compared with the other groups. In terms of “precision,” conventional impressions with silicone or polyether were significantly superior to digital impressions with either scanner. Silicone and polyether showed no statistically significant difference. Hence, while making the impression for implant prostheses, these factors need to be considered.

González Menéndez et al 49 compared the angular deviation of two, four, and six dental implants placed with and without the use of parallel copings, using cone-beam computed tomography scans and 3D implant-planning software. They reported that the use of parallelization copings resulted in lower deviation when two or four implants were included in the impression. However, this technique was not effective in cases involving a greater number of implants. Furthermore, Osman et al 50 evaluated the accuracy of impression of a simulated area restored with an implant-retained fixed partial denture, using the open- and closed-tray implant impression techniques and studied the effect of implant position angulation, parallelism, and implant systems (Straumann, SIC Invent, and Osstem). They found that there was no significant difference between open and closed, although better results were obtained for the open-tray techniques. With nonparallel implants, the open-tray technique provided a better result compared with the closed-tray technique. Papaspyridakos et al 13 compared the 3D accuracy of splinted versus unsplinted impression techniques and determined the optimal clinically undetectable misfit. They mentioned that for the external connection, an acceptable 3D misfit in implant-fixed prosthesis ranges from 59 to 72 μm, resulting in an acceptable fit clinically. In addition, they found that the splinted technique showed more accurate master casts than the unsplinted one in edentulous jaws, which is similar to our study. In our research, technique D with parallel implant placement showed the best results. In addition, a recent study shows that the dynamic computer-assisted implant surgery provides similar accuracy of the 3D implant position and parallelism between two implants compared with static and dynamic computer-assisted implant surgery. 51 52

With the rise of digital dentistry, digital impressions using intraoral scanners have been adopted in implant dentistry, but the accuracy is a major issue in long-span arches. 21 In addition, the difficulty in scanning the soft tissue between implants in edentulous arches remains a major issue. There are various methods to increase the accuracy of the full arch impression. Greater accuracy is seen when the implants are placed in bounded edentulous areas with guided surgery using computer-aided design/computer-aided manufacturing-manufactured surgical guides. 8 In digital impression, the successful stitching of images is important for accuracy, and this process is directly related to both the scanning scope and the interimplant distance. 53 54 A study by Kim et al 55 mentioned that an artificial landmark in the long edentulous area can improve the trueness and precision of the intraoral scanners. The auxiliary geometric devices attached to implant scan bodies via flowable composite can increase 3D spatial recognition of soft tissues. 56 Similarly, Arikan et al 19 suggested that the use of auxiliary geometric appliances yields increased scanning accuracy. In addition, they suggested that the frameworks fabricated using the traditional splinted open-tray technique were more reliable compared with those frameworks from digital impressions.

There are some limitations in this research study. This study investigated only conventional impressions. Second, the limited sample of dental implants due to time and financial constraints, and the findings should be interpreted carefully. In the future, the conventional impression technique can be compared with the digital impression techniques.

## Conclusion

The dental implants can be placed parallel or tilted and the implant impression can be made via splinted versus unsplinted impression techniques. The impression technique affects the accuracy of the implant impression. Within the limitations of this study, the impression copings splinted with dental floss and self-cure acrylic resin showed the highest distortion. There seems to be no advantage clinically in splinting transfer impression copings with self-cure acrylic resin in the case of parallel implants. Further clinical studies are needed.
